# Decoding Host Cell–Virus Interactions: Translational Advances in Antiviral Immunity and Therapeutics (4th Edition)

**DOI:** 10.3390/v18030286

**Published:** 2026-02-27

**Authors:** Anupam Mukherjee, Parikshit Bagchi

**Affiliations:** 1 ICMR-National Institute of Virology, Pune 411001, India; 2 Department of Molecular Microbiology, Washington University School of Medicine in St. Louis, St. Louis, MO 63110, USA

Viruses remain among the most formidable biological threats to global health, continually challenging existing therapeutic and preventive strategies through rapid evolution and immune escape. Central to viral pathogenesis is the dynamic interaction between invading pathogens and host cellular systems, encompassing innate immune sensing, inflammatory signaling, cellular stress responses, and adaptive immunity. These processes collectively determine infection outcomes and provide critical entry points for therapeutic intervention.

Since its inception in 2020, the Special Issue series *Host Cell–Virus Interaction* has served as a dedicated platform for disseminating advances in virus–host biology. Across four editions over the past five years, this initiative has attracted contributions from researchers worldwide, resulting in more than 50 published articles addressing diverse aspects of viral lifecycle regulation, immune evasion, and antiviral development. The present fourth edition features twelve peer-reviewed contributions (five Research Articles, five Reviews, one Communication, and one Opinion), reflecting both conceptual progress and translational innovation in the field.

Several original research studies in this edition address immune dynamics and host determinants of viral infection. Brangel et al. report a WHO-coordinated, multi-laboratory comparison of SARS-CoV-2 neutralization assays, demonstrating strong concordance between live-virus and pseudovirus platforms and providing a standardized framework for monitoring emerging variants and evaluating vaccine-induced immunity [1]. Bekbossynova et al. reveal persistent depletion of naïve CD8^+^ T cells in SARS-CoV-2–infected individuals, extending into convalescence and highlighting long-term immune imprinting that may influence susceptibility to secondary infections and vaccine responsiveness [2]. Li et al. identify extensive interferon-driven circular RNA remodeling in porcine alveolar macrophages, positioning IFN-ω5–regulated circRNA networks as emerging modulators of antiviral immunity [3]. Wang et al. engineer a recombinant rabies virus expressing GitrL, demonstrating enhanced dendritic cell activation and accelerated neutralizing antibody responses, thereby offering a promising strategy for improving live-attenuated vaccine efficacy [4]. Nosik et al. uncover subtype-specific effects of estradiol and progesterone on Toll-like receptor expression during HIV-1 infection, emphasizing how host hormonal status and viral genetic diversity jointly shape immune responses and viral replication [5].

Complementing these mechanistic studies, five Review Articles provide broader perspectives on host–virus interactions and therapeutic opportunities. Sreepangi et al. examine host-driven ubiquitination pathways exploited by vector-borne RNA viruses, highlighting E3 ligases as actionable targets for broad-spectrum antiviral strategies [6]. Banerjee et al. synthesize current knowledge on γ-herpesvirus-mediated oncogenesis, detailing genetic, epigenetic, and immune mechanisms underlying Epstein–Barr virus and Kaposi’s sarcoma-associated herpesvirus-driven malignancies, with implications for antiviral and anticancer therapeutics [7]. Pereira Santos et al. focus on the HPV E5 oncoprotein in head and neck cancers, outlining its roles in immune evasion and therapy resistance and identifying E5 as a potential molecular target in HPV-associated disease [8]. Alanazi et al. adopt a translational lens to host–virus interactions across major RNA viruses, emphasizing host-directed antivirals and immune modulation as next-generation strategies to overcome resistance associated with virus-centric treatments [9]. Suneesh et al. review advances in human organoid models, illustrating how three-dimensional stem-cell-derived systems enable physiologically relevant studies of viral tropism, immune signaling, and antiviral screening, thereby accelerating precision virology and therapeutic discovery [10].

The Communication by Shishova et al. reveals how encephalomyocarditis virus subverts the unfolded protein response by inducing IRE1 phosphorylation while suppressing XBP1 mRNA splicing, uncovering a viral strategy to uncouple cellular stress signaling and promote replication [11]. Extending conceptual frameworks in innate immunity, Das et al., in their Opinion article, propose a novel “tyrosine kinase axis” involving Src-family kinases, Syk, BTK, and EGFR that governs Toll-like receptor and STING activation, linking viral nucleic acid sensing to spatially regulated interferon responses and identifying new opportunities for therapeutic immune modulation [12].

Collectively, the contributions in this Special Issue highlight several themes of immediate translational relevance, including identification of host dependency and restriction factors, refinement of vaccine platforms, discovery of host-directed antiviral targets, and deployment of advanced human-relevant model systems for therapeutic screening. Multiple studies emphasize that targeting conserved host pathways, rather than viral components alone, may provide durable antiviral efficacy with a higher barrier to resistance. Advances in immune profiling, ubiquitination biology, organoid technologies, and kinase-regulated innate sensing presented here directly inform the development of next-generation vaccines, immunomodulators, and broad-spectrum antivirals. Importantly, these host-centric strategies also support pandemic preparedness by enabling rapid therapeutic repurposing against emerging viral threats. Together, these insights reinforce the growing paradigm that integrating molecular virology with translational immunology is essential for accelerating the pipeline from mechanistic discovery to clinical intervention.

As Editors of this Special Issue, we are pleased to present this fourth edition of *Host Cell–Virus Interaction*, which builds upon the success of previous editions and reinforces the value of sustained, multidisciplinary inquiry into virus–host biology. We sincerely thank all authors for their valuable contributions and the reviewers for their critical evaluations. We also acknowledge the MDPI editorial team for their continued support. We hope that this collection will serve as a valuable resource for researchers and clinicians alike and will stimulate further advances toward translational solutions for viral diseases.
